# Dual-Frequency Non-invasive Monopolar Radiofrequency for Periorbital Tightening: Introduction of Novel Small Treatment Tips

**DOI:** 10.7759/cureus.101187

**Published:** 2026-01-09

**Authors:** Guy Erlich, Eliran Dahan, Ron Skorochod, Yoram Wolf

**Affiliations:** 1 Plastic Surgery, Hillel Yaffe Medical Center, hadera, ISR; 2 Medicine, Rappaport Faculty of Medicine, Technion - Israel Institute of Technology, Haifa, ISR; 3 Family Medicine, Meuhedet Health Maintenance Organization (HMO), Haifa, ISR; 4 Plastic Surgery, G2 Aesthetics, Hadera, ISR; 5 Medicine, G2 Aesthetics, Hadera, ISR; 6 Plastic Surgery, Hillel Yaffe Medical Center, Hadera, ISR; 7 Medicine, Rappaport Faculty of Medicine, Technion – Israel Institute of Technology, Haifa, ISR

**Keywords:** dual-frequency radiofrequency, eyebrow elevation, monopolar radiofrequency, periorbital rejuvenation, upper eyelid tightening

## Abstract

Periorbital aging involves progressive alterations in the dermis, superficial musculoaponeurotic system (SMAS), and ligamentous support, leading to eyebrow descent, upper-eyelid redundancy, and periorbital rhytids. Noninvasive monopolar radiofrequency (NMRF) is designed to address tissue laxity across multiple anatomical layers, yet conventional small applicators are limited by their shallow penetration, reducing their effectiveness. A novel dual-frequency NMRF platform (XERF, Cynosure-Lutronic, South Korea), incorporating two newly engineered small applicators, was developed to address this depth constraint. This report aims to describe our standardized approach to periorbital rejuvenation using XERF’s novel small applicators, with the primary objectives of improving eyebrow position and achieving upper eyelid tightening. At the time of reporting, across all procedures performed to date, no unexpected or persistent adverse reactions were observed. Limited early imaging demonstrated the expected immediate tissue contraction, and qualitative impressions suggested interval improvements in eyebrow position, upper-eyelid redundancy, and periorbital rhytids at three months; however, these observations are anecdotal and not presented as quantitative outcomes. The author assumes that the novel small applicators may facilitate more controlled vector-based energy delivery, and that the dual-frequency deep mode may theoretically reduce the superficial penetration limitations of conventional small tips. As this manuscript is intended solely to outline a standardized procedural protocol, these assumptions should not be interpreted as evidence-based conclusions. Objective and subjective outcome measures are being collected in an ongoing prospective study. In conclusion, the XERF dual-frequency NMRF system, equipped with its novel small tips, appears to deliver safe, precise, and reproducible nonsurgical periorbital rejuvenation.

## Introduction

Periorbital aging encompasses progressive multilayered alterations involving the dermis [1,2], the superficial musculoaponeurotic system (SMAS) [2-4], and the ligamentous support network [2,5,6]. Collectively, these changes disrupt fat distribution, weaken structural support, and reduce resistance to gravitational forces [2,5,7], leading clinically to lateral eyebrow, eyelid, and canthal descent, along with increased prominence of forehead and lateral canthus wrinkles [2].

Noninvasive monopolar radiofrequency (NMRF) delivers thermal energy efficiently across multiple tissue layers, engaging both the dermis and deeper supporting structures [8,9]. Clinical studies have demonstrated that NMRF can produce measurable periorbital tightening, eyebrow elevation and reduction of periorbital wrinkles [10,11]. Adverse events with NMRF are generally infrequent. In a retrospective observational study of 290 patients, severe pain occurred in 11.49% of treatment sessions, second-degree burns in 2.7%, persistent erythema lasting >24 hours in 1.22%, and persistent edema in 0.68% (>24 h) and 0.53% (>48 h). Rarer complications included third-degree burns with scarring, headache appearing 1 week post-treatment, fat atrophy, atrophic scarring, return-pad burns, and neuralgia with facial palsy, each reported in ≤0.26% of sessions [12]. When performed with appropriate technique and conservative parameters, NMRF can be delivered safely without injury to the eyelids or ocular structures [13].

In addition to temperature and impedance monitoring, NMRF delivery in a safe and effective manner requires a perpendicular tip angle with full skin contact [14]. Because forehead convexity and frontal sinus width vary by age, sex, and race [15], achieving consistent contact in this region can be difficult. Smaller tips were therefore developed to accommodate these anatomical variations, enhancing precision while preserving safety [13]. The depth of NMRF penetration is predominantly determined by the geometry and surface area of the active electrode. Smaller electrodes concentrate current density superficially, producing precise but shallow heating [16,17], which may benefit fine rhytids. However, the limited depth can be insufficient for lifting and tightening procedures that require contraction of deeper supporting tissues, such as the periorbital SMAS.

A novel dual-frequency NMRF system (XERF, Cynosure-Lutronic, South Korea) delivers 6.78 MHz and 2 MHz simultaneously, producing immediate thickening and shortening of collagen bundles in the superficial dermis as well as in the superficial and deep fascial layers [18,19]. This dual-frequency platform may overcome the depth limitations typically associated with conventional NMRF small tips. This report aims to outline our standardized approach to periorbital rejuvenation using NMRF.

## Technical report

This technical report presents our standardized approach to periorbital rejuvenation using the XERF system (Cynosure-Lutronic, South Korea) and its newly developed small-surface applicators, effector 10 (1 cm²; 10 × 10 mm) and effector 5 (0.5 cm²; 10 × 5 mm). The objective of this report was to describe the procedural methodology, while a formal outcome assessment is being conducted concurrently in an ongoing parallel study.

Adult patients seeking nonsurgical periorbital rejuvenation are being prospectively considered for treatment as part of an actively recruiting study. Eligible participants are required to comply with procedural instructions, attend scheduled follow-up visits, and provide written informed consent following discussion of the anticipated effects of NMRF and its potential adverse events or complications. Exclusion criteria included pregnancy or breastfeeding; the presence of implanted electronic devices, including but not limited to pacemakers, defibrillators, neurostimulators, or cochlear implants; and the presence of metal hardware that could interfere with NMRF current flow. Additional exclusions comprised contraindications to NMRF based on manufacturer recommendations or clinician judgment, active dermatologic disease within the treatment area, a history of keloid formation or impaired wound healing, uncontrolled systemic conditions that could alter tissue response, and any recent or planned periorbital procedures within six months prior to treatment or during the three-month follow-up period that could confound evaluation.

Treatment protocol

Before treatment began, a dispersive return pad (grounding pad) was applied according to manufacturer guidelines, and patients were positioned supine. The periorbital region was thoroughly cleansed of any makeup or topical products, after which a generous layer of ultrasound gel was applied to the skin to enhance energy conduction and facilitate smooth, controlled movement of the treatment tip during the procedure. Since the procedure is generally well tolerated, the use of topical anesthetic cream was optional and guided by patient preference. According to the manufacturer’s guidelines, corneoscleral protective lenses are recommended when energy is delivered directly toward the eye on the eyelid. In our protocol, energy delivery to the eyelid was confined to the infra-eyebrow region and along the orbital rim, without directing energy toward the globe; therefore, protective lenses were not used.

**Figure 1:**
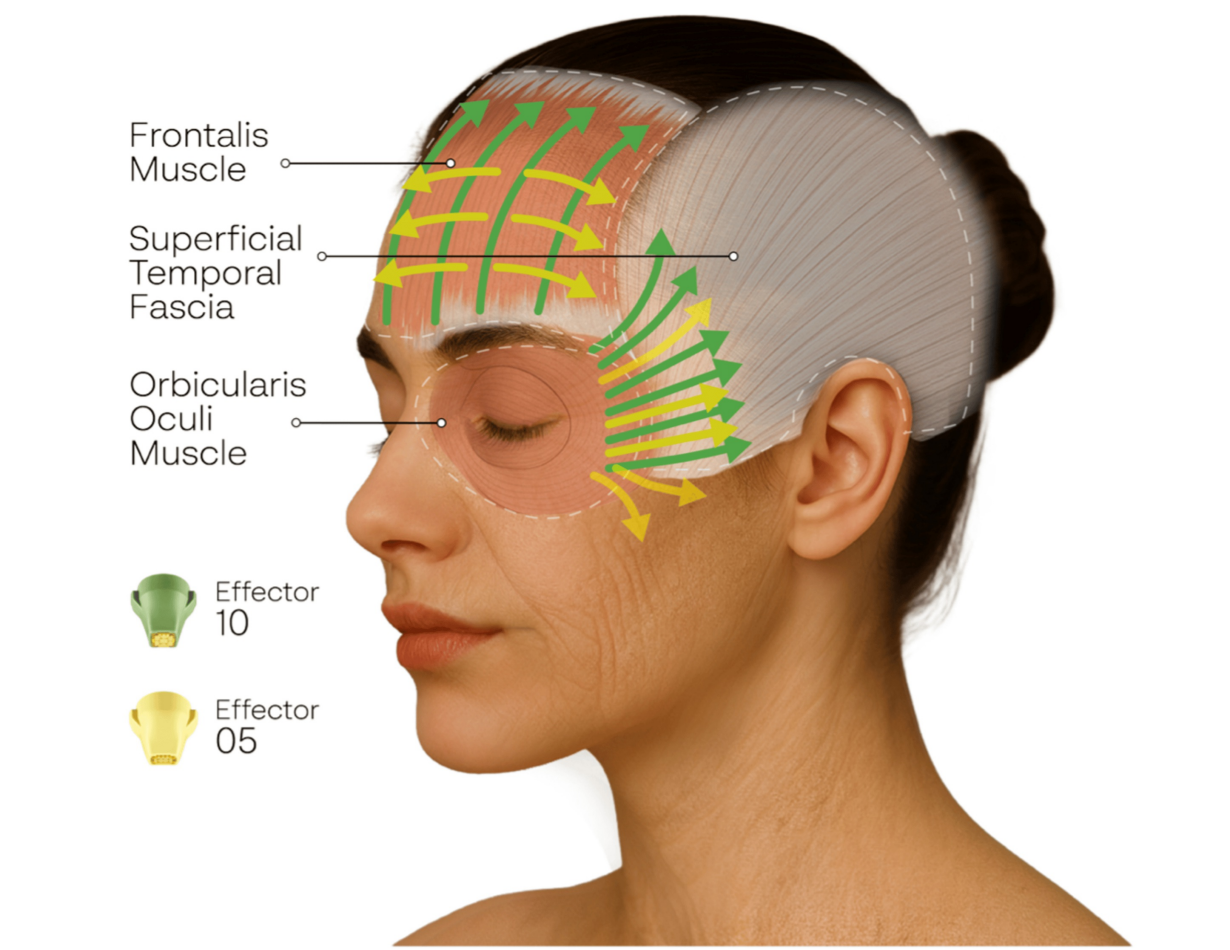
Anatomical illustration depicting the periorbital treatment strategy using the dual-frequency XERF NMRF system. The diagram demonstrates the spatial relationship of the frontalis, orbicularis oculi, and superficial temporal fascia, which represent key structural targets for thermal modulation. Green vectors indicate the upward and lateral energy-delivery directions used with Effector 10, while yellow vectors depict the more focused passes applied to static rhytids along their natural wrinkle axes. Created by Cynosure-Lutronic on author's request.

Treatment was performed in deep mode, delivering simultaneous 6.78 MHz and 2 MHz radiofrequency. Effector 10 was used first. After impedance synchronization at the temple, 100 pulses per side were delivered using lateral and upward vectors. Treatment is initiated along the orbital rim with the applicator angled away from the globe while maintaining perpendicular skin contact. Gentle upward traction was applied to stabilize and elevate the upper-eyelid skin, enabling controlled treatment of the infra-eyebrow skin and the contour of the orbital rim while maintaining a safe distance from the ocular surface. Pulses were applied evenly across the forehead, temporal, and periorbital regions, with all vectors oriented away from the eye and initiated outside the orbital rim. Effector 5, optimized for precise treatment of focal areas, was subsequently used to address static rhytids. The E5 applicator was aligned with each wrinkle’s natural axis, and 50 pulses per side were applied uniformly across all targeted lines (Figure 1). The primary therapeutic objectives were to achieve measurable eyebrow elevation and clinically meaningful tightening of the upper-eyelid complex.

Each treatment line began with continuous-motion preheating to optimize tissue conductivity, followed by stamping along the same vector. Energy delivery was adjusted according to patient tolerance, targeting a moderate discomfort level corresponding to a score of 3 on a standardized four-point pain scale, where 1 indicates no pain, 2 mild pain, 3 moderate pain, and 4 severe pain. This degree of tolerance corresponded to device energy settings in the range of levels 4 to 6. Integrated contact cooling (ICD) was maintained at level 1 throughout the procedure. Although well tolerated in our cohort, the ICD level could be adjusted up to level 3 to enhance patient comfort if required (Table 1). Patients were continuously monitored for any signs of discomfort or cutaneous reactions, including erythema, edema, or sensations of excessive heat or pain.

**Table 1:** Treatment parameters for periorbital NMRF using XERF’s novel small applicators Energy levels are reported according to device output settings and were titrated to patient tolerance, targeting moderate discomfort (pain score 3 on a 4-point scale), corresponding to energy levels 4–6. Integrated contact cooling (ICD) was maintained at level 1 throughout the procedure and could be increased up to level 3 to enhance patient comfort if required. Pulse numbers indicate the total number of pulses delivered per side for each applicator.

Effector Type	Energy (Level)	Cooling (ICD Level)	Pulses/side
Effector 10 (1 cm²; 10 × 10 mm)	4-6	1	100
Effector 5 (0.5 cm²; 10 × 5 mm)	4-6	1	50

Post-treatment instructions and follow-up assessment

Immediately after the procedure, patients received standardized post-treatment instructions. They were advised that transient cutaneous responses, including erythema, warmth, mild edema, or tingling, may occur and generally resolve within several hours. Patients were instructed to avoid hot showers, saunas, strenuous physical activity, and potentially irritant topical agents for 48 hours. In addition, patients were advised to apply a non-irritating moisturizing cream to maintain adequate skin hydration, along with immediate initiation of broad-spectrum photoprotection (SPF 50), which was maintained throughout the three-month follow-up period. Standardized clinical imaging was performed using the LifeViz® system (QuantifiCare, France), a validated three-dimensional imaging platform designed for reproducible facial surface capture. The system enables high-resolution acquisition of facial topography under controlled lighting and positioning conditions, allowing consistent documentation of contour changes over time [20]. Imaging was obtained at baseline, immediately post-procedure, and during scheduled follow-up assessments at one and three months.

## Discussion

**Figure 2:**
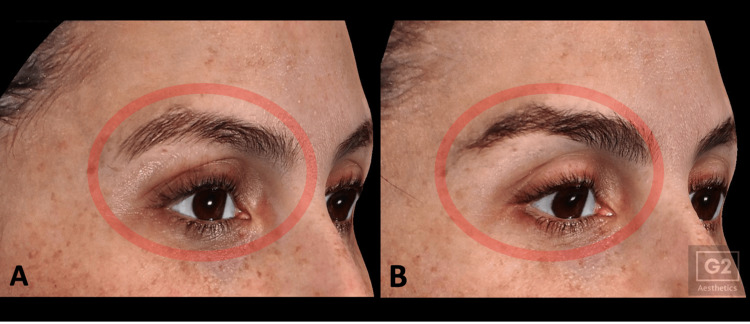
Periorbital changes following a single NMRF treatment session Periorbital images of a 39-year-old female patient at baseline (A) and at three-month follow-up (B) following a single NMRF treatment session (XERF, Deep mode; E10 100 pulses per side and E5 50 pulses per side) demonstrating improved eyebrow elevation and enhanced upper-eyelid contour.

At the time of writing, all procedures have been well tolerated, with no complications observed. Mild transient erythema occurred in some cases and resolved spontaneously within several hours. No episodes of prolonged edema exceeding 24 hours, burns, contour irregularities, or pigmentary alterations were noted. Imaging consistently demonstrated the expected immediate post-treatment tissue responses, and follow-up imaging at one and three months showed qualitative improvements in eyebrow position, upper-eyelid redundancy, and periorbital fine lines (Figure 2).

This technical report describes the author’s experience using the novel small applicators (effector 10 and effector 5) of the dual-frequency XERF NMRF system in the anatomically complex periorbital region. Their reduced surface area is assumed to enhance procedural precision and maintain more consistent contact across curved anatomical surfaces, which may facilitate controlled vector-based energy delivery while reducing the likelihood of unintended orbital exposure. Although smaller tips might prolong treatment duration, by requiring more pulses per area, the operator did not perceive the procedure as longer. Ergonomically, the smaller applicators were more comfortable to handle and did not necessitate frequent adjustments to maintain full and uniform contact with the skin. The deep-mode dual-frequency output may help address the limited penetration typically associated with small applicators, potentially allowing more uniform heating of deeper fascial layers.

Early qualitative impressions noted by the author on LifeViz® imaging, such as possible improvement in brow position and upper-eyelid skin redundancy, appear consistent with the expected tissue responses of this technique. The protocol represents a time-efficient, anatomy-adapted approach to periorbital treatment, intended to be performed with minimal patient discomfort and limited downtime.

This technical report describes a standardized procedural protocol and does not present quantitative outcome data. Objective and subjective assessments, including brow height measurements, wrinkle grading scales, and patient- and evaluator-reported improvement scores, are being collected as part of an ongoing prospective study. Accordingly, the observations described here are preliminary and reflect the author’s impressions only; the blinded results of the ongoing study will be required to determine the magnitude and durability of the treatment response.

## Conclusions

The dual-frequency NMRF system, incorporating newly developed small tips (effector 10 and effector 5), may provide a precise and anatomically adaptable approach for periorbital treatment. Procedures performed to date have been well tolerated, and early imaging has subjectively indicated potential improvements in eyebrow position, upper-eyelid redundancy, and periorbital fine lines; however, these impressions are preliminary and have not been validated. The small applicators are assumed to enhance stability and enable controlled vector-based energy delivery in the periorbital region, while the deep-mode dual-frequency output may mitigate the limited penetration typically associated with conventional small tips. These assumptions will be examined further as objective data from the ongoing prospective study become available. Overall, the protocol is intended to offer a safe, efficient, and reproducible method for periorbital rejuvenation with minimal discomfort and limited downtime.
